# Efficacy based ginger fingerprinting reveals potential antiproliferative analytes for triple negative breast cancer

**DOI:** 10.1038/s41598-020-75707-0

**Published:** 2020-11-05

**Authors:** Lihan Zhao, Manali Rupji, Ishita Choudhary, Remus Osan, Shobhna Kapoor, Hong-Jie Zhang, Chunhua Yang, Ritu Aneja

**Affiliations:** 1 Department of Biology, Georgia State University, Atlanta, GA 30303 USA; 2 School of Chinese Medicine, Hong Kong Baptist University, Kowloon Tong, Hong Kong; 3 Biostatistics and Bioinformatics Shared Resource, Winship Cancer Institute of Emory University, Atlanta, GA 30322 USA; 4 Department of Math and Stats, Georgia State University, Atlanta, GA 30303 USA; 5 Department of Chemistry, Indian Institute of Technology Bombay, Powai, Mumbai, Maharashtra 400076 India; 6 Institute for Biomedical Sciences, Georgia State University, Atlanta, GA 30303 USA

**Keywords:** Cancer, Breast cancer, Cancer therapy, Drug discovery, Drug screening

## Abstract

Ginger (*Zingiber officinale*) is one of the most widely consumed dietary supplements worldwide. Its anticancer potential has been demonstrated in various studies. However, ginger roots obtained from different geographical locations showed extensive variability in their activities, mainly due to differences in the levels of bioactive compounds. Here we evaluated the effect of these differences on the anticancer activity of ginger by performing efficacy-based fingerprinting. We characterized the fingerprint profiles of 22 ginger samples using liquid chromatography-mass spectroscopy, followed by a principal component analysis (PCA) and pearson correlation analysis. We also evaluated the anti-proliferative effects (IC_50_) of these samples on triple-negative breast cancer cells using the MTT assays. The supervised PCA identified a subset of analytes whose abundance strongly associated with the IC_50_ values of the ginger extracts, providing a link between ginger extract composition and in vitro anticancer efficacy. This study demonstrated that variation in the ginger fingerprint profiles resulting from differences in their chemical composition could have a significant impact on efficacy and bioactivity of ginger extracts. Also, this first-of-a-kind efficacy-based fingerprinting approach proposed here can identify potent anticancer candidates from the ginger fingerprint without the need for isolating individual components from the extracts.

## Introduction

Natural remedies (also known as natural medicines) have been indispensable for cancer therapy, as over 50% of current anticancer agents are isolated from natural resources, including plants, marine organisms, and microorganisms^1^. Though whole extracts are believed to be superior to isolated single compounds, the therapeutic potential of natural medicines has been controversial^2^. The chemical heterogeneity in natural medicines obtained from different origins is a major concern, limiting their extensive use in the clinics^3^. Unlike synthetic drugs, natural extracts are composed of hundreds or even thousands of compounds. The concentration of bioactive compounds in natural extracts varies immensely. For example, the consistency of plant extracts depends on several environmental factors, including the area of cultivation, climate, planting time, harvest time, age at harvesting, and post-harvesting storage^4^. The compositional diversity of phytochemicals present in the natural extracts is further affected by the processing and extraction method, processing time, sample particle size, and sample to solvent ratio, among other factors^5,6^.

Traditionally, one or more chemical markers are quantitated to allow evaluation of the quality control (QC) of natural medicines. However, this process is insufficient, as the quality assessment is based solely on a few constituents out of a complex mixture of bioactive compounds contained in a natural extract. With the development of various analytical techniques in recent years, chromatographic fingerprint methods have emerged as an attractive approach for identification and QC of herbal products^7^. A chromatographic fingerprint of an extract is the chromatographic pattern of its chemical components or pharmacologically-active constituents. The main advantage of these new methods is the fact that they provide information on the complete chemical profile of natural extracts, and has, therefore, become a standard QC method endorsed by the World Health Organization (WHO) for herbal medicines^8^. Thus, with the help of chromatographic fingerprints, a more accurate quality assessment of herbal medicines, regardless of variations in chemical constituents, has become possible^9^.

Ginger (*Zingiber officinale* Roscoe) has been widely used in traditional Chinese, Ayurvedic, and Tibb-Unani herbal medicines since ancient times, demonstrating a wide range of benefits, including the alleviation of nausea, appetite loss, motion sickness, and pain^10,11^. Notably, ginger extracts have been reported to exhibit anticancer effects in various in vitro assays and xenograft mouse models^12^. Our group and others have demonstrated antitumor effects of whole ginger extracts or isolated ginger phenolic compounds against prostate cancer, both in vitro and in vivo^13–15^. Given the potential clinical relevance of ginger, a robust QC method is necessary for accurately and reliably evaluating the quality of ginger products. Chromatographic fingerprint methods have already been used in the QC of ginger extracts^16,17^. Ginger samples from India, Malaysia, Thailand, Vietnam, and different parts of China were fingerprinted, and analytical approaches, such as principal component analysis (PCA), were utilized to discriminate the origins of ginger^16,17^. However, these studies did not assess potential correlations between ginger’s chemical composition and its biological activity, thus failed to provide meaningfully relevant information.

**Figure 1:**
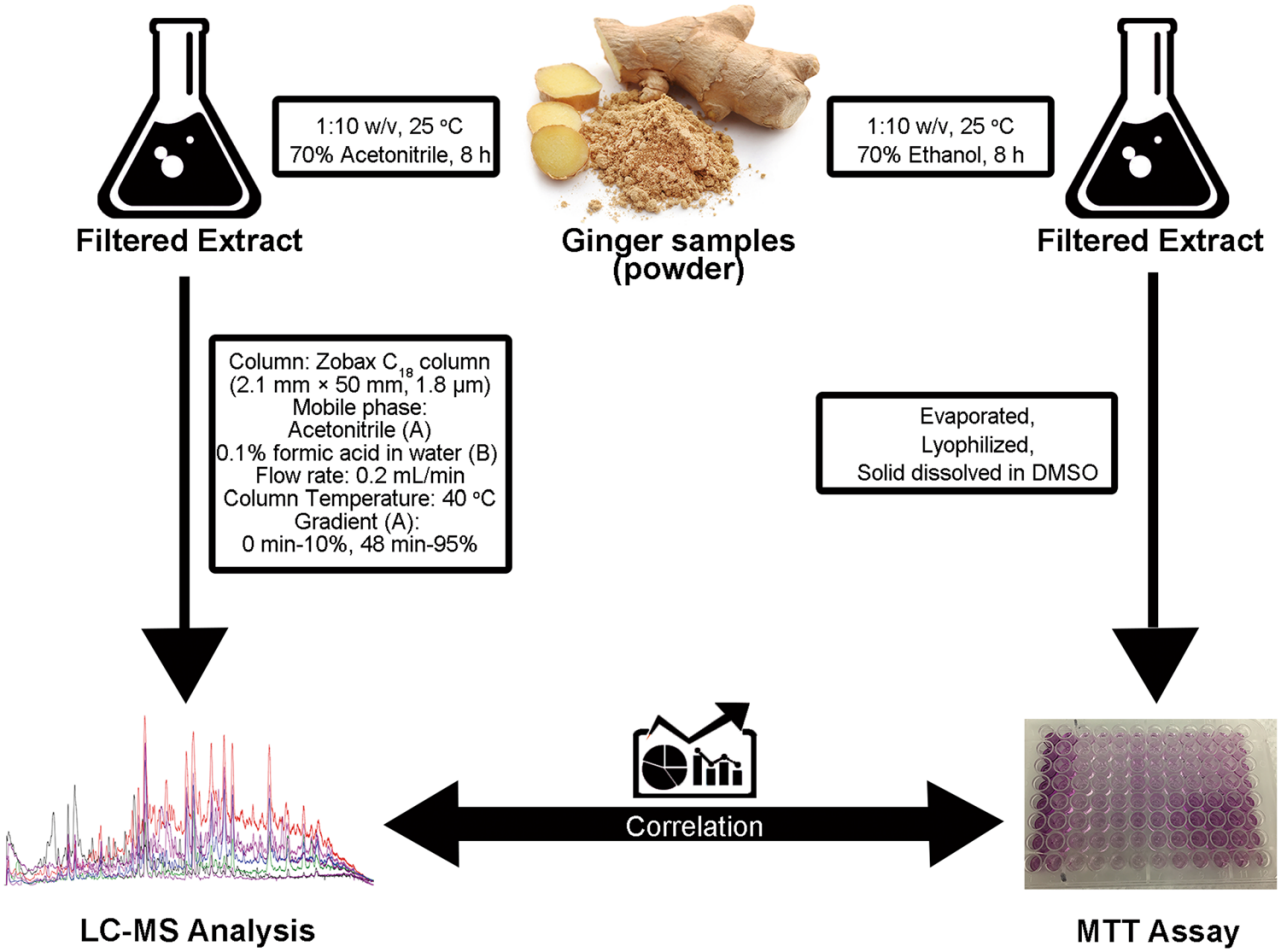
Schematic overview of the efficacy-based ginger fingerprinting approach.

Triple-negative breast cancer (TNBC), which lacks the expression of the estrogen receptor (ER), progesterone receptor (PR), and excess amplification of human epidermal growth factor receptor 2 (HER2), is the most aggressive subtype of breast cancer^18,19^. Certain ginger phenolic compounds have been shown to exert promising antitumor effects against TNBC, by inducing apoptosis and inhibiting metastatic dissemination of cancer cells, providing a strong rationale for the use of ginger in therapeutic approaches in TNBC^20^. However, a major challenge preventing the wide use of ginger in the clinics is the lack of methods to accurately determine its bioactive constituents. The approach traditionally used for drug discovery from medicinal herbs usually involves (1) activity-guided fractionation of the plant extract, (2) lead compound isolation and identification, (3) in vitro and in vivo efficacy evaluation and (4) lead optimization^21^. However, the first three steps are tedious and usually lead to redundancy in isolated compounds or loss of activity after isolation. To counteract these issues, we utilized an analytical approach to determine the relationship between the concentration of bioactive analytes and their anti-proliferative capacity in ginger extract. This strategy led us to create an “efficacy fingerprint” of ginger extract by correlating the chemical fingerprint with the antiproliferative activity using statistical approaches. This strategy can ultimately identify the most potent anticancer components from ginger’s chemical fingerprint profile (Fig. 1), which can be further developed as mechanism-based therapeutics to treat TNBC.

## Results

### Ginger extract preparation and chemical profiling

**Figure 2:**
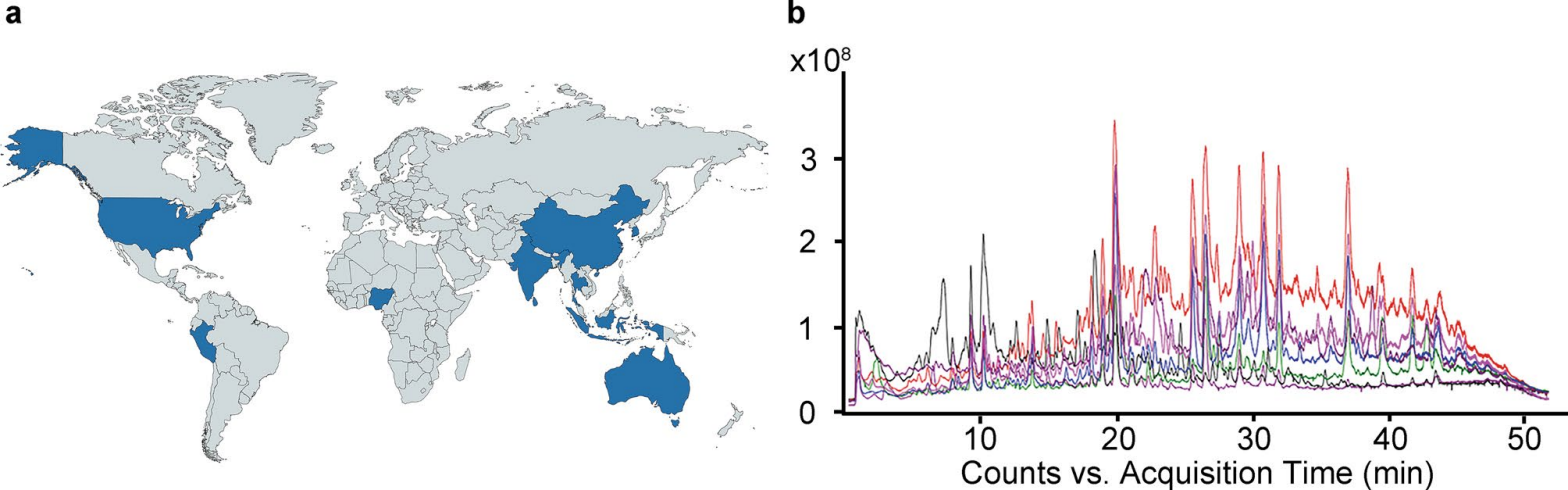
Ginger extract origins and chemical profiling. (**a**) Origins (marked with dark blue color) of 22 ginger samples used for our analysis (figure created with mapchart.net). (**b**) Representative chromatogram acquired by positive ion mode LC–MS.

We first extracted analytes from ginger samples of different geographical origins with 70% acetonitrile overnight (Fig. 2a, and Supplementary Table S1). Next, we established a high-performance liquid chromatography-mass spectrometry (LC–MS) method to characterize the chemical composition of all the ginger extracts by using both a positive and a negative ion mode. Our LC–MS data suggested that the positive ion mode LC–MS provided higher sensitivity. Our method validation showed that the relative standard deviations (RSDs) of precision, stability, and repeatability were no more than 2% for both retention time and peak area (Supplementary Fig. S1), indicating that the analysis method used was reliable and reproducible. Next, fingerprint profiling of ginger extracts was performed by high-performance LC–MS (Fig. 2b and Supplementary Fig. S2). Based on the retention time (RT), MS, and MS/MS spectra from positive ion mode LC–MS, we identified 68 analytes with a signal intensity of over 10^6^. These analytes were annotated by retention time, m/z, and MS/MS spectrum information (Supplementary Table S2), and were subjected to quantitative analysis.

### The chemometric analysis showed similarity among ginger samples

**Figure 3:**
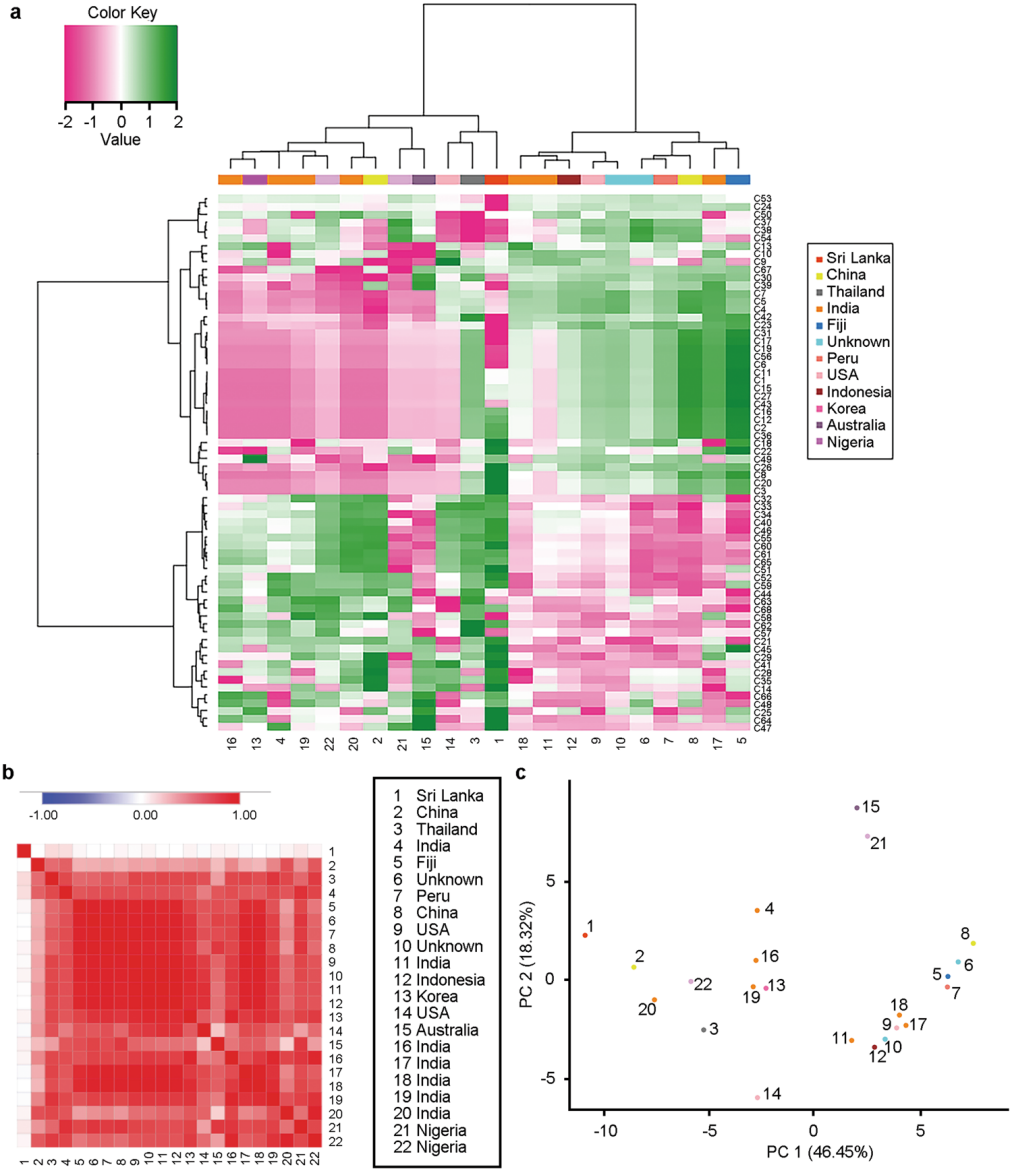
Chemometric analysis of 22 ginger samples across 68 major components. (**a**) Heatmap showing the ginger samples (columns) and the 68 major components (rows), after row normalization and Ward’s clustering with Manhattan distance. Green indicates higher peak areas, whereas magenta represents lower peak areas for the components. Heatmap was created using NOJAH tool (https://bbisr.shinyapps.winship.emory.edu/NOJAH.html)^25^. (**b**) Pearson correlation analysis between 22 ginger samples based on the 68 components. Red indicates a positive correlation; blue indicates a negative correlation, while white indicates no correlation. The correlation plot was created using the Morpheus tool (https://software.broadinstitute.org/morpheus/). (**c**) PCA analysis results. PC, principal component. PCA plot was created in RStudio using the 'stats' package.

Median normalization is the most commonly used normalization method when internal standards are unavailable. Hence, prior to chemometric analysis, the chromatographic data acquired from ginger extracts were median-normalized using NOREVA (normalization and evaluation of MS-based metabolomics data, https://idrblab.cn/noreva2017/, https://server.idrb.cqu.edu.cn/noreva/)^22–24^. Inspection of the relative log abundance (RLA) plots along with distribution of each ginger sample based on box plots determined that a median normalization was appropriate. Data distribution before and after normalization is shown in Supplementary Fig. S3a and b. To evaluate if ginger samples and/or components showed similarities based on the fingerprint profile, we performed hierarchical clustering analysis (HCA). As shown in the heatmap of Fig. 3a, the ginger samples formed two sample clusters (column dendrogram), which appeared to be defined by two distinct sets of components (row clusters). However, there was no association between the ginger sample clusters and the country of origin (Fisher's exact p = 0.175).

Pearson correlation analysis revealed that the samples were very similar (Fig. 3b), since when samples were compared based on their country of origin, no striking differences were apparent. The only sample that was distinct and showed much lower similarity to the rest was the ginger sample from Sri Lanka. This finding could be attributed to the unique components (such as C1–C3) that were present in this sample but not in the rest, in addition to the differential abundance of components common to all ginger samples.

We performed PCA analysis to evaluate whether the ginger samples cluster into distinct groups based on their composition and if the clustering is associated with their origin. Similarly, to what HCA showed, PCA analysis indicated that the ginger samples form distinct clusters. When analyzed by the country of origin, no specific clustering was apparent, suggesting that origin was not the major contributor to the differences between the ginger samples, consistent with HCA and correlation analysis (Fig. 3c). Therefore, the country of origin alone cannot be used as a factor to determine the quality and bioactivity of ginger; additional factors need to be considered.

### Anti-proliferative activity of ginger extracts

**Figure 4:**
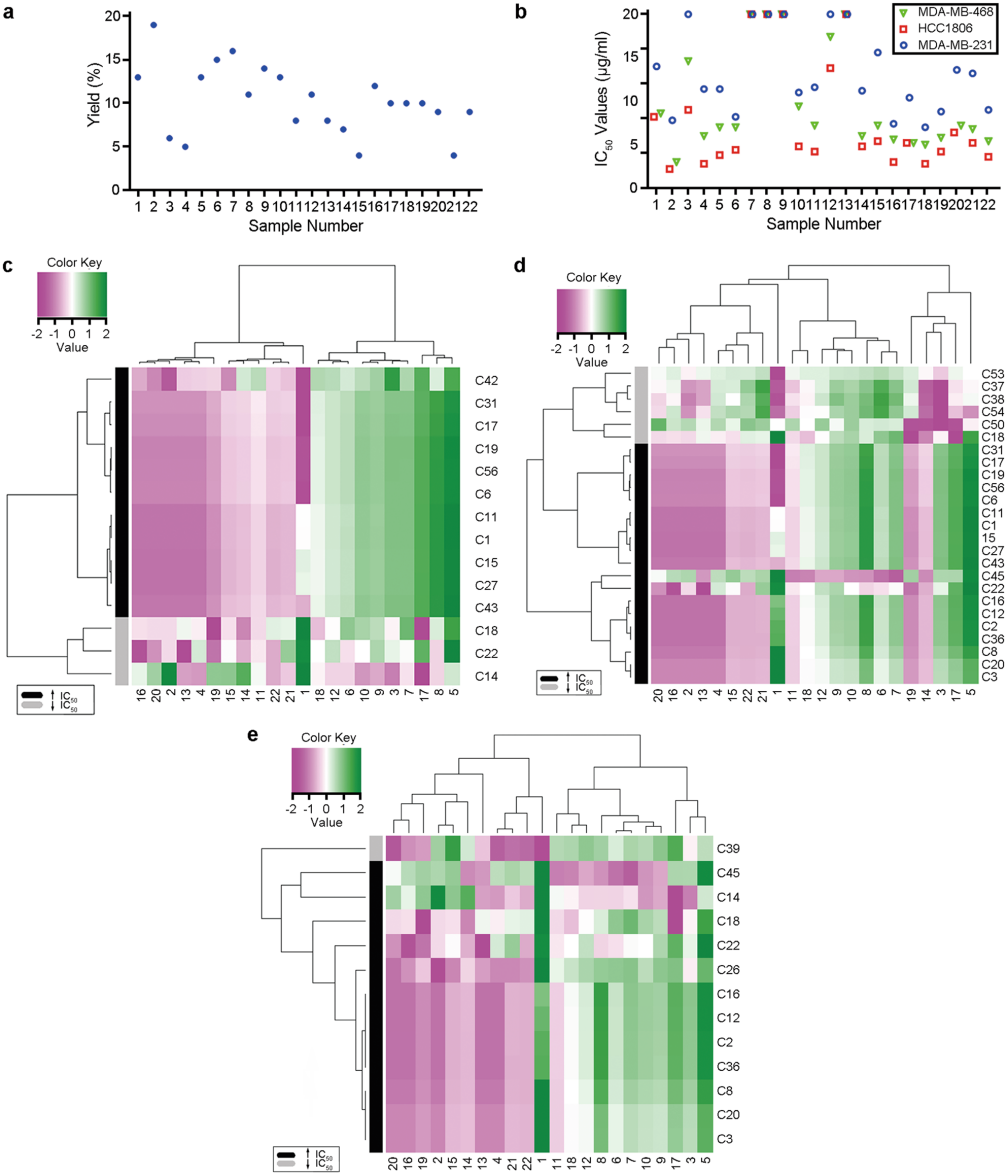
Anti-proliferative effects of ginger extracts and their association with their chemical composition. (**a**) The yield of 22 ginger sample extracts prepared for the MTT assay. (**b**) MTT assay results revealed the anti-proliferative effects of ginger samples in MDA-MB-231, HCC1806, and MDA-MB-468 cells. (**c–e**) Heatmaps of the components found to have the most potent effects on IC_50_ values (either increasing or decreasing) in MDA-MB-231 (**c**), HCC1806 (**d**), and MDA-MB-468 (**e**) cell lines. Green color indicates higher peak areas, whereas magenta represents lower peak areas for the components, where the peak areas have been normalized.

The extraction of ginger samples gave an average yield of 11%, ranging between 4 and 19% (Fig. 4a and Supplementary Table S1). We used 3-(4, 5-dimethylthiazol-2-yl)-2, 5-diphenylterazolium bromide (MTT) assay to evaluate the anti-proliferative activity of 22 ginger extracts in three human breast cancer cell lines (MBA-MD-231, HCC1806, and MBA-MD-468). The most potent anti-proliferative effect was observed with the ginger sample from Nature’s mojo (ginger sample 2) against HCC1806 cells, with an IC_50_ value of 11 µg/mL (Fig. 4b and Supplementary Table S1).

### Statistical analysis correlated components with anti-proliferative effects

Semi-supervised principal component analysis, Gene Associations with Clinical (GAC)^26,27^, was used to assess the effect of individual components on the proliferation of TNBC cells, based on their IC_50_ values in the three different TNBC cell lines. GAC identified 14, 25, and 13 most effective components (Supplementary Table S3) based on their IC_50_ values in MDA-MB-231 (p = 0.043 for the first principal component), HCC1806 (p = 0.014 for the first principal component), and MDA-MB-468 (p = 0.004 for the first principal component). A total of 29 components were identified to exert anti-proliferative effects in either of the cell lines, while two components in all three cell lines. Most components had consistent effects on anti-proliferative activity across the three cell lines except three components (C14, C18, and C22), indicating a cell-specific behavior. HCA of the significant components was able to cluster components based on their ability to decrease the anti-proliferative effects of ginger extracts (to increase IC_50_) or to increase their anti-proliferative capacity (to decrease IC_50_) in each of the cell lines (Fig. 4c–e).

### Compound identification

**Figure 5:**
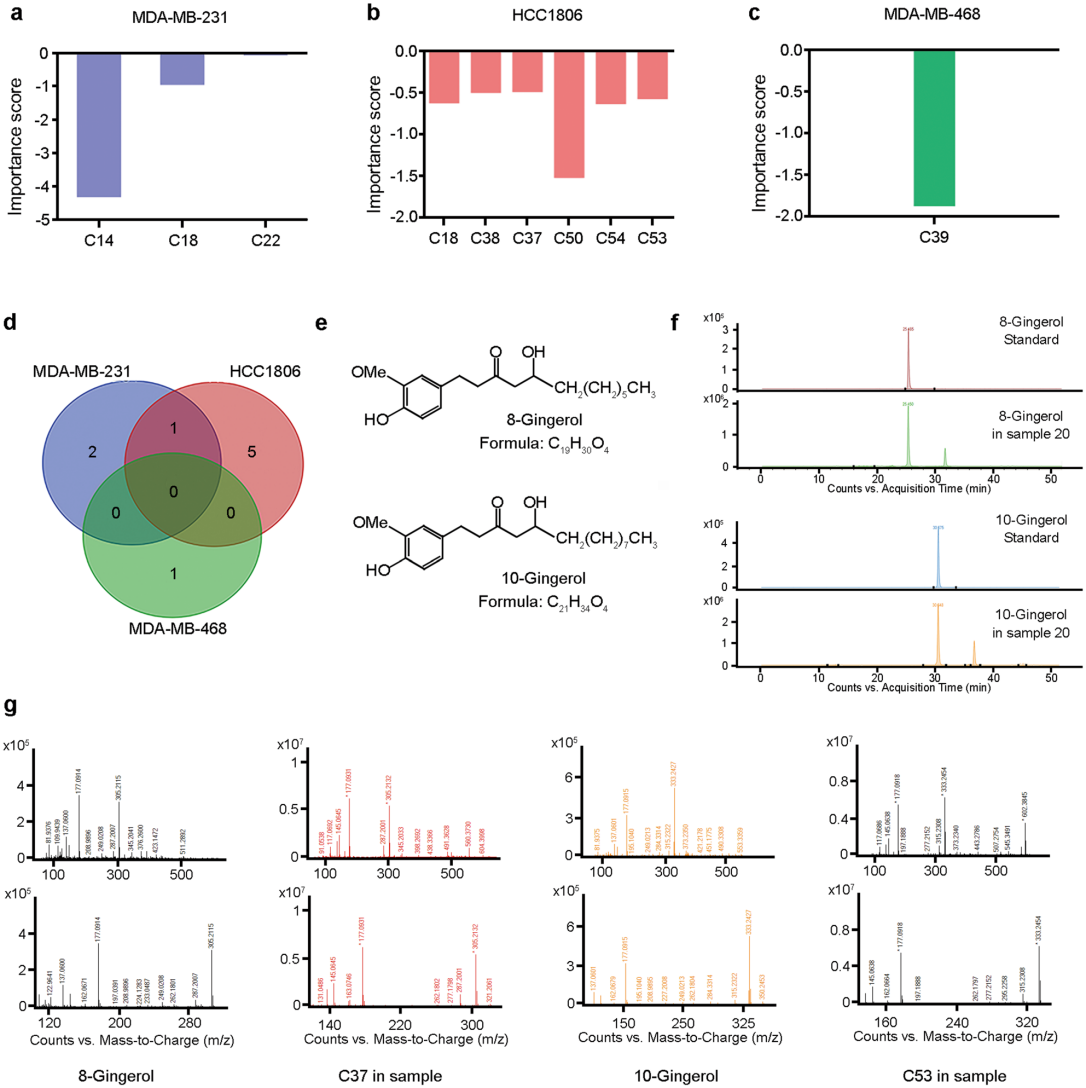
Identification of components that enhance the anti-proliferative effects of ginger extracts. (**a–c**) The importance scores of the analytes that were the most potent in decreasing the IC_50_ values in MDA-MB-231 (**a**), HCC1806 (**b**), and MDA-MB-468 **(c)**. (**d**) Venn diagram showing the number of components identified to enhance the anti-proliferative effects of ginger extracts in TNBC cell lines. (**e**) The structure of 8-gingerol and 10-gingerol molecules. (**f**) Chromatograph comparing the retention time of 8-gingerol and 10-gingerol standards to their retention time in ginger sample 20. (**g**) The positive MS/MS spectrum information of 8/10-gingerols in authentic standards and in ginger sample 2.

We summarized the analytes that enhanced the anti-proliferative effects of the ginger extracts, as indicated by the decreased IC_50_ values (Fig. 5a–c and Supplementary Fig. S4 for analytes with the opposite effects). We identified three, six, and one analyte/s that enhanced the ginger-mediated growth inhibition in MDA-MB-231, HCC1806, and MDA-MB-468 cells, respectively (Fig. 5d). C14 and C50 were the most potent compounds inhibiting the growth of MDA-MB-231 and HCC1806, respectively, while only one analyte (C39) was able to inhibit the growth of MDA-MB-468 cells. The analyte C18 was a common compound inhibiting the growth of both MDA-MB-231 and HCC1806. We further analyzed analytes by assessing their mass spectra (Supplementary Table S4). We also compared their retention times and MS/MS data with those of the standard compounds and confirmed that the analyte C37 was 8-gingerol, while C53 was 10-gingerol (Fig. 5e–g). Moreover, we also predicted the structures of other analytes using their MS/MS data (Supplementary Fig. S5). Both C18 and C22 had similar *m/z* values to 6-paradol ([ESI]^+^ 261.1880 and 261.1881, cal: 261.1849, [M + H-H_2_O]^+^); thus, they were postulated to have a molecular formula of C_17_H_26_O_3_. They also both appeared to produce several peaks at *m/z* 137 and 163, which represent characteristic product ions of 6-paradol with cleavage at C_1_-C_2_. However, since the retention time of C18 and C22 were different from the 6-paradol standard, we hypothesize that they might be isomers of 6-paradol. Additionally, both C38 and C39 showed similar *m/z* values to 8-gingerol, ([ESI]^+^ 305.2166 and 305.2142, cal: 305.2111, [M + H-H_2_O]^+^), and were postulated to have a molecular formula of C_19_H_30_O_4_, with a class characteristic product ion of *m/z* 177. Similarly, C54 had an *m/z* value of 333.2482, which is close to 10-gingerol, C_21_H_34_O_4_ (cal: 333.2424, [M + H- H_2_O]^+^), with a class characteristic product ion of *m/z* 177.

### Spiking confirmed the anti-proliferative effects of the identified compounds

To confirm the anti-proliferative effects of 8-gingerol and 10-gingerol, we performed MTT assay with three groups of ginger samples, namely, (1) ginger sample containing low abundance of 8- and 10-gingerol, (2) ginger sample containing high abundance of 8- and 10-gingerol, and (3) ginger sample containing low abundance of 8- and 10-gingerol, spiked with requisite amounts of 8- and 10-gingerol to simulate the group 2 ginger sample.

**Figure 6:**
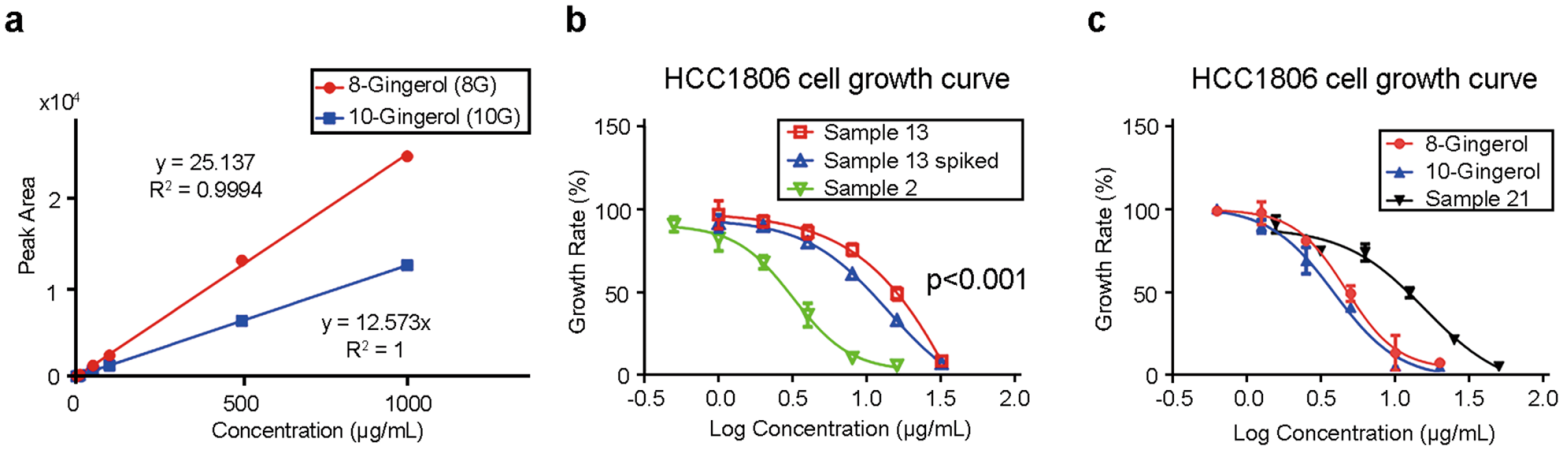
In vitro cell proliferation spike-and-recovery assay confirming the ability of 8-gingerol and 10-gingerol to enhance the anti-proliferative effects of ginger against HCC1806 cell line. (**a**) The calibration curve for 8-gingerol and 10-gingerol used to determine the concentration difference between ginger samples. The concentration of injected solution was converted into µg/mL in ginger samples. (**b**) The growth curve of ginger sample 13, ginger sample spiked with 8- and 10-gingerols, and ginger sample 2 at various concentrations (log) against HCC1806 cell line. The overall p-value between the three groups (ginger sample 13, spiked 13, and 2) is < 0.001. Additionally, there is a statistically significant difference between each comparison, ginger samples 13 and 2, spiked 13 and 2, and spiked 13 and 13 (each adjusted p < 0.001). (**c**) The anti-proliferative effects of 8-gingerol and 10-gingerol as a single compound, compared to that of ginger sample 21 against HCC1806 cell line.

To determine the concentrations of the analytes of interest in the ginger samples, we analyzed 8-gingerol and 10-gingerol standards by LC–UV–MS and obtained two calibration curves using LC–UV (wavelength of 282 nm) peak area with R square of 0.9994 (for 8-gingerol) and 1 (for 10-gingerol), respectively (Fig. 6a). Subsequently, we assessed the concentration of 8-gingerol and 10-gingerol in ginger samples. Since GAC indicated that 8-gingerol and 10-gingerol are the crucial compounds that enhanced the anti-proliferative effects of ginger samples in HCC1806 cells, we performed the MTT assay and found that the ginger sample 2 had the most potent anti-proliferative effects in this cell line, while ginger sample 13 was the least potent. According to the calibration curve, the difference in concentrations of 8-gingerol and 10-gingerol in these two ginger samples was 0.71 µg/mg for 8-gingerol and 2.73 µg/mg for 10-gingerol (see Supplementary Table S5 for absolute quantification for all peaks).

For the cell proliferation spike-and-recovery assay, the amount of the difference in concentrations of 8-gingerol and 10-gingerol between ginger samples 2 and 13 was added to ginger sample 13 (as the third group) to test the effects of spiking on its IC_50_ value. The IC_50_ values decreased after spiking (from more than 100 µg/ml to 44 µg/ml), indicating an increased anti-proliferative effect (Fig. 6b). The overall p-value between the three groups (ginger sample 13, spiked 13, and 2) is < 0.001. Additionally, there is a statistically significant difference between each comparison, ginger samples 13 and 2, spiked 13 and 2, and spiked 13 and 13 (each adjusted p < 0.001). Since 8- and 10-gingerols are perhaps not the only selective compounds contributing to decrease of the IC_50_ values of ginger samples, the decreased IC_50_ value is not as low as that of ginger sample 2 (around 10 µg/ml). However, the effects of spiking 8- and 10-gingerols are already noticeable. To further investigate the effects of 8- and 10-gingerols in HCC1806 proliferation, we observed the growth curves versus the concentrations (log) of 8- and 10-gingerol are to the left compared to the curve of ginger sample 21, which had the highest concentration of 8- and 10-gingerol, indicating that 8- and 10-gingerols are potent anti-proliferative compounds, as shown in Fig. 6c. These findings highlight that our established chemometric and statistical analyses are able to identify potential anti-proliferative compounds from ginger fingerprint profiles.

## Discussion

A critical barrier to the development of natural medicines as cancer therapeutics is the difficulty of controlling the quality of such remedies, as many environmental factors affect their composition, efficacy and bioactivity^4^. Current QC methods focus merely on evaluating the concentration of the most abundant constituents; therefore, they provide only limited information with regards to the efficacy and toxicity of natural medicine. Hence, the development of a new holistic approach that could assess the bioactive chemical components of a natural medicine and determine the relationship between particular components and the efficacy of the product is of high importance. Herein, using ginger extract as a prototype example, we describe an approach that provides a framework to establish a relationship between the chemical fingerprint profile and the efficacy profile of 22 ginger extracts to create an efficacy fingerprint (or signature). This innovative approach identifies ginger components that are essential for the in vitro anti-proliferative capacity against TNBC.

Several extraction methods for natural products have been established over the years. Methanol has been previously used to prepare ginger extracts for LC–MS analysis^16,17^. In our study, we prepared ginger extracts using acetonitrile as the extraction solvent, based on the fact that acetonitrile not only shares close polarity with methanol but also is fully compatible with the mobile phase system of the LC–MS. Previous studies revealed that ginger extracts from different origins could be differentiated based on their chromatographic fingerprint profile^16,17^. However, PCA could not cluster the ginger samples based on their origins in our study. This might be due to the lack of geoclimatic diversity in our samples collected from each country. Correlation analysis showed that all the samples shared a high similarity, and many common peaks were present in all ginger samples. However, the concentrations of individual ginger compounds varied significantly among ginger samples of different origin. These findings portray the inherent compositional variability of natural medicines and thus, relying on a QC method that merely depends on the chemical fingerprints yields very limited information on its efficacy.

We next investigated the anti-proliferative activity of 22 ginger extracts in three TNBC cell lines, and their IC_50_ values were used for statsitical analysis. The ginger sample 2 had the most significant anti-proliferative effects, with an IC_50_ value of 11 µg/mL in HCC1806 cell lines. To our surprise, ginger samples 7, 8, 9, and 13 did not show any anti-proliferative effects in any of the cell lines tested, even when used at 100 µg/mL, while the samples 3 and 12 exhibited only moderate growth inhibition. This high variability in the anti-proliferative effects of ginger extracts can be attributed to differences in their compositional profiles.

Subsequently, we performed structure identification of the analytes selected by GAC, with a particular emphasis on the analytes that exhibited potent anti-proliferative effects. Two compounds (8-gingerol and 10-gingerol) were confirmed by comparing their retention time with those of standards. 10-gingerol has been previously reported to exert anti-tumor activity in TNBC, by inhibiting tumor cells proliferation, invasion, and metastasis, as well as by inducing apoptosis, both in vitro and in vivo^20,28,29^. Additionally, 10-gingerol demonstrated potent anti-tumor effects on non-TNBCs and other types of cancer^30,31^. To the best of our knowledge, 8-gingerol has not been previously reported to have anti-tumor activity in TNBC; thus, herein, we provide for the first-time evidence that suggests the potential use of 8-gingerol in TNBC treatment. Our study also suggested C18 and C22 and attractive lead compounds. Although C18 and C22 share identical MS and MS/MS spectra with 6-paradol, their retention time differs from that of 6-paradol; we, therefore, speculate that they are isomers of 6-paradol. According to a compound novelty searching (SciFinder, https://sso.cas.org), C18 and C22 might be novel compounds, and their potential use in TNBC treatment merits further investigation. We noticed that C14 was the most potent compound that inhibited the growth of MDA-MB-231 cells. We tentatively speculate it as PE (phosphoethanolamine) or PC (phosphocholine) which is abundant in plant membrane, but further efforts are needed for the confirmation and identification of this component.

In conclusion, this study showed that high variability among the ginger fingerprint profiles resulting from differences in chemical composition could have a significant impact on efficacy and bioactivity of ginger extracts. Correlating the chemical components as shown by the chemical fingerprint to the anti-proliferative efficacy of ginger offers an excellent approach for identifying potent anticancer candidates using their fingerprint profiles. This approach might also be useful for establishing a robust QC method that offers reliable and accurate insights into the quality of ginger, reducing the amount of time needed to identify lead compounds in herbal medicines.

## Methods

### Materials and reagents

Twenty ginger samples from different origins were purchased from different vendors through Amazon.com, and two ginger samples were collected from Sagamu and Zaria districts of Nigeria (details provided in Supplementary Table S1). All samples were in the form of dry powder. Six-gingerol (6G, ≥ 98% purity), 8-gingerol (8G, ≥ 95% purity), 10-gingerol (10G, ≥ 95% purity), ethyl cinnamate, cinnamyl acetate, 8-shogaol, and MTT were purchased from Sigma Aldrich (St. Louis, MO, USA). Six-paradol was purchased from Adooq (Irvine, CA, USA), while pinolenic acid was sourced from Santa Cruz (Dallas, TX, USA). Deionized water was prepared using a Milli-Q purification system (Millipore, Billerica, MA, USA). LC–MS grade acetonitrile, methanol, and formic acid were purchased from Sigma Aldrich (St. Louis, MO, USA). Ethanol was purchased from Decon Laboratories, Inc. (King of Prussia, PA, USA). Dimethyl sulfoxide (DMSO) was purchased from Fisher Scientific (Pittsburgh, PA, USA).

### Ginger extract preparation

Ginger samples were extracted with 70% acetonitrile for fingerprinting profiling, or with 70% ethanol for antiproliferative assay. Both extraction processes were conducted at room temperature for approximately 8 h with a powder to solvent ratio of one to ten (w/v). For ginger fingerprint profiling, ginger extracts were filtered using a 0.22 µm filter before LC–MS analysis. For MTT assay, ginger extracts were filtered using a 0.22 µm filter before solvent volume reduction by rotary evaporation, and the samples were dried by lyophilization.

### LC–MS

Chromatographic analysis was performed using the Agilent 1290 Infinity II LC system (Agilent, Santa Clara, CA, USA). A Zobax C_18_ column (2.1 mm × 50 mm, 1.8 μm; Agilent) was used for separation; the mobile phase consisted of acetonitrile (gradient of 10% at 0 min and 95% at 48 min) (A) and 0.1% formic acid in water (B). The flow rate was 0.2 mL/min with a column temperature at 40 °C. The mass spectrometry was performed using the Agilent Q-TOF 6545 system (Agilent, Santa Clara, CA, USA), using the following conditions: nebulizer pressure, 35 PSIG; capillary voltage, 3500 V; fragmentor voltage, 175 V; drying gas flow, 12 L/min; drying gas temperature, 320 °C; sheath gas flow, 11 L/min; sheath gas temperature, 350 °C. The mass range was recorded from *m/z* 50 to 1700 Dalton. Data acquisition was performed using the MassHunter Workstation (Agilent, Santa Clara, CA, USA). The fingerprints of ginger samples were prepared using the MassHunter Profinder (Agilent, Santa Clara, CA, USA). A solvent(reagent) blank was analyzed together with ginger samples to trace sources of artificially introduced contamination. The raw LC–MS chromatograph was extracted, and a collection of major components (defined by their signal intensity of 10^6^ by height) was used for further analysis. The components were annotated by retention time, m/z, and MS/MS spectrum information, and the values of retention time and peak area were averaged and normalized using the online tool NOREVA (https://server.idrb.cqu.edu.cn/noreva/)^22–24^. Data imputation was performed using the default settings in NOREVA, to account for at most 20% non-missing within in each sample and additionally 30% non-missing for each component^22^.

### Chemometric analysis

#### HCA and heatmap generation

HCA is a multivariate analysis method that can be used to divide samples into groups. Heatmap generation and HCA of the ginger samples were performed using the online tool NOJAH (NOt Just Another Heatmap, https://bbisr.shinyapps.winship.emory.edu/NOJAH/)^25^. For generating a heatmap, row values were z-score normalized, and hierarchical clustering was performed using Ward’s method with Manhattan distance, to allow for the identification of the similarities and differences in the components of the ginger samples.

#### PCA

PCA was performed using NOREVA, to assess the distribution of ginger samples into potential clusters based on their country of origin. This technique can reduce the dimensions of the original dataset by using a subset of underlying factors to represent the correlations without losing important information.

#### Pearson correlation analysis

To evaluate the relationship between different ginger samples and identify potential connections, a 1-pearson correlation analysis across all components was performed using Morpheus (https://software.broadinstitute.org/morpheus/).

### MTT assay

MTT assays were performed to assess the effects of ginger samples on cell viability and proliferation. MDA-MB-231 (ATCC HTB-26), HCC1806 (ATCC CRL-2335), and MDA-MB-468 (ATCC HTB-132) TNBC cells were purchased from American Type Culture Collection (Manassas, VA, USA) in December 2014. Cell lines were periodically confirmed negative for mycoplasma contamination using PCR assays and passaged according to the supplier’s instructions. Cells were seeded in 96-well microculture plates (3500–5000 cells per well). After 24 h, the cell culture medium was replaced by fresh medium with or without different concentrations of ginger extracts (1.5625, 3.125, 6.25, 12.5, 25, 50, 75, and 100 μg/mL), and cells were incubated for 48 h at 37 °C with 5% CO_2_. After 48 h, the medium containing ginger extracts was aspirated, 100 μL of medium containing 0.5 mg/mL MTT was added to each well, and the plates were incubated for another 3 h in the dark. Subsequently, the solution was removed, and 100 µL of DMSO was added to each well. After 15 min of shaking, the absorbance at 570 nm was read on a microplate spectrophotometer (MD Plate Reader, Perkin Elmer). Samples from four vendors (Simply organic, Great America Spices Co., Thrive market, and Rom America) did not show anti-proliferative activity in any of the cell lines, even at 100 μg/mL. These four samples were marked as “> 100” in tables and given an estimated value of 200 μg/mL for further statistical analysis.

### Statistical analysis

GAC, a web-based application that uses a semi-supervised principal component (SuperPC) approach, was employed to access the components’ effects on the proliferation of three representative TNBC cell lines^26,27^. The peak area values were inputted as the gene expression levels, while the IC_50_ values as the quantitative outcome. To determine which components are significantly associated with the anti-proliferative outcome, SuperPC was performed using the best fitting model, a 60–40 split for training and validation, and a fivefold cross-validation approach with up to 200 iterations. The resulting list of components and their importance scores demonstrated the contribution of different components to the IC_50_ values of ginger extracts. Positive scores suggested an increased anti-proliferative activity, whereas negative scores indicated attenuated anti-proliferative activity of the ginger extract.

### Compound identification and quantification

Original chromatograms and mass spectra of compounds of interests were retrieved and submitted to an online metabolomics database metlin (https://metlin.scripps.edu/), to identify their chemical formulas and structures, followed by a comparison between their MS/MS information with those recorded in literature and mass spectrometry database MoNA (with GNPS library included, https://mona.fiehnlab.ucdavis.edu/). Several commercialized standards were purchased and tested for their LC–MS behavior. The retention times and mass spectra of standards were used to verify the postulated compounds. For the verified compounds, a calibration curve (from 0.5 to 1000 µg/mL) was created, and their concentration was determined in the 22 ginger samples using the calibration curve. Compounds that showed retention times different from the standards were tentatively presented with their MS/MS fragmentation in supplementary material.

### Spike-and-recovery assessment of ginger samples

Spike-and-recovery experiments were performed by adding a certain amount of isolated compounds in ginger samples (ginger sample 13) that showed less potent anti-proliferation activity and contained a low amount of identified compounds. The amount of compound added was determined by the difference of identified compounds’ concentration between the ginger samples with high and low anti-proliferation abilities. In this case, 89 µL of 8-gingerol and 343 µL of 10-gingerol in methanol (1 mg/mL) were vacuum evaporated and dissolved in 1 mL ginger sample 13 (10 mg/mL) as the stock solution prepared for MTT assay. This stock solution was further twofold series diluted and then added into the media of HCC1806 cell lines to test their anti-proliferation activity. The IC_50_ values were obtained using MTT assays, after treating cells with ginger samples with or without the compound of interest. The differences between groups and log concentrations for the growth rate curves were calculated using a 2-way ANOVA using the 'car' R package and Tukey's HSD post hoc multiple correction test was used to estimate pairwise differences.

## Supplementary information

Supplementary Information.
