# Supplementary material for: Efficacy based ginger fingerprinting reveals potential antiproliferative analytes for triple negative breast cancer
Source: Sci Rep. 2020 Nov 5;10:19182. doi: 10.1038/s41598-020-75707-0 (PMC7644756; doi:10.1038/s41598-020-75707-0)
Supplement: Supplementary file 1 — Supplementary Information. [file 41598_2020_75707_MOESM1_ESM.pdf]

Efficacy based ginger fingerprinting reveals potential antiproliferative analytes for triple negative breast cancer

Lihan Zhao [1, 2], Manali Rupji [3], Ishita Choudhary [1], Remus Osan [4], Shobhna Kapoor [5], Hong-Jie Zhang [2], Chunhua Yang [6,*], and Ritu Aneja [1,*]

[1]Department of Biology, Georgia State University, Atlanta, GA 30303, USA
[2]School of Chinese Medicine, Hong Kong Baptist University, Hong Kong
[3]Biostatistics and Bioinformatics Shared Resource, Winship Cancer Institute of Emory University, Atlanta, GA 30322, USA
[4]Department of Math and Stats, Georgia State University, Atlanta, GA 30303, USA
[5]Department of Chemistry, Indian Institute of Technology Bombay, Powai, Mumbai, Maharashtra 400076, India
[6]Institute for Biomedical Sciences, Georgia State University, Atlanta, GA 30303, USA
*****cyang16@gsu.edu; raneja@gsu.edu

Supplementary Table S1. Country of origin, percent yield and MTT anti-proliferation data for various ginger extracts.

Sample No.	Ginger samples with origin	Yield (%)	MDA-MB-231	HCC1806	MDA-MB-468
				IC_{50} (µg/mL)	
**1**	Frontier (Sri Lanka)	13	70	41	43
**2**	Nature's Mojo (China)	19	39	11	15
**3**	Spicy World (Thailand)	6	>100	45	73
**4**	Organic Veda (India)	5	57	14	30
**5**	Wakaya (Fiji)	13	57	19	35
**6**	Thrive Market (unknown)	14	41	22	35
**7**	Simply Organic (Peru)	11	>100	>100	>100
**8**	Starwest Botanicals, Inc. (China)	15	>100	>100	>100
**9**	Great America Spice Co. (USA)	16	>100	>100	>100
**10**	Ava’s Essential Co. (unknown)	13	55	24	47
**11**	Anthony's (India)	8	58	21	36
**12**	San Antonio (Indonesia)	11	>100	69	87
**13**	Rom America (Korea)	8	>100	>100	>100
**14**	Gerbs (USA)	7	56	24	30
**15**	Super Sprout (Australia)	4	78	27	36
**16**	McCormick Gourmet (India)	12	37	15	28
**17**	Jiva Organics (India)	10	52	26	26
**18**	Blue Lily Organics (India)	10	35	14	25
**19**	Indus Organics (India)	10	44	21	29
**20**	International Spice (India)	9	68	32	36
**21**	Sagamu (Nigeria)	4	66	26	34
**22**	Zaria (Nigeria)	9	45	18	27

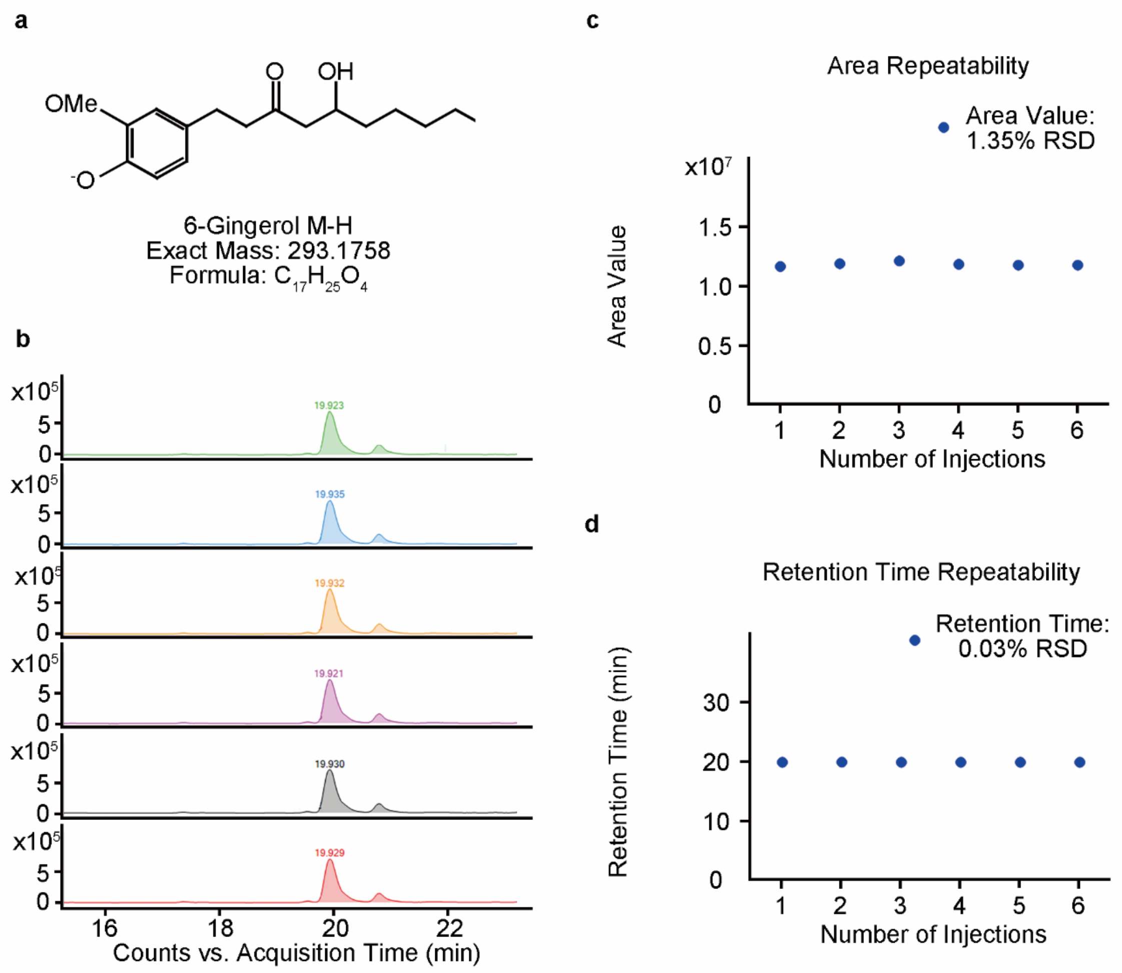

Supplementary Figure S1. Method validation of ginger extracts' LC-MS analysis. **(a)** The structure of 6-gingerol. **(b)** The chromatography of 6-gingerol standard acquired by LC-MS for 6 replicates. **(c-d)** Area and retention time repeatability of 6-gingerol.

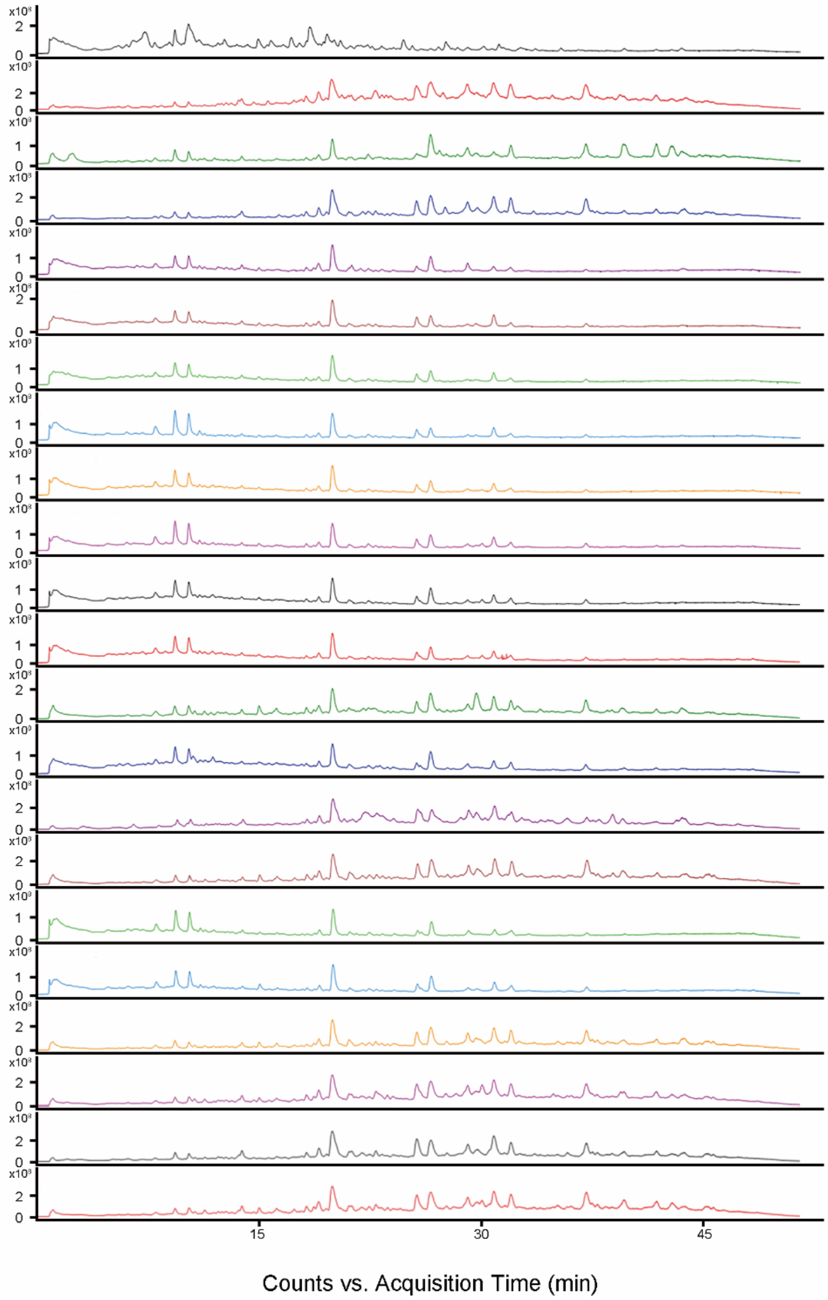

Supplementary Figure S2. The chromatography of 22 ginger samples acquired by LC-MS, positive mode.

Supplementary Table S2. The retention time of selected analytes along with the peak area of 22 ginger samples.

			LC-MS peak area for each ginger sample																					
**Peak No.**	Mass	RT	1	2	3	4	5	6	7	8	9	10	11	12	13	14	15	16	17	18	19	20	21	22
**C1**	578.165	7.05	88872893	0	0	0	0	0	0	0	0	0	0	0	0	0	0	0	0	0	0	0	0	0
**C2**	543.213	7.32	1.25E+08	0	0	0	0	0	0	0	0	0	0	0	0	0	0	0	0	0	0	0	0	0
**C3**	675.255	7.47	2.75E+08	0	0	0	0	0	0	0	0	0	0	0	0	0	0	0	0	0	0	0	0	0
**C4**	565.421	8.01	85727303	29149901	37029539	35542670	40741735	43269961	44243516	83055545	51151035	76898013	48769727	52443941	37562397	43665362	30035105	36639145	48809171	48038426	35836042	35253096	36419417	35553462
**C5**	678.509	9.35	2.01E+08	79501940	91848459	78148461	1.17E+08	1.22E+08	1.27E+08	2.3E+08	1.45E+08	2.13E+08	1.35E+08	1.45E+08	89872088	1.28E+08	86480015	87438355	1.42E+08	1.37E+08	84716525	83190099	97922155	79117272
**C6**	562.17	10.14	55878726	0	0	0	0	0	0	0	0	0	0	0	0	0	0	0	0	0	0	0	0	0
**C7**	791.586	10.27	1.38E+08	66449424	66952007	61618304	1.12E+08	1.08E+08	1.09E+08	1.94E+08	1.11E+08	1.88E+08	1.15E+08	1.23E+08	73160224	1.1E+08	65872237	61979406	1.27E+08	1.3E+08	53778905	57931564	69645583	60296119
**C8**	555.213	10.38	1.72E+08	0	0	0	0	0	0	0	0	0	0	0	0	0	0	0	0	0	0	0	0	0
**C9**	293.198	10.64	0	1354190	4890579	3163351	945742.3	1867325	2573353	781240.6	5108527	8380205	13096885	10371095	22412879	71693307	1886868	9420493	4672839	9209380	22560875	42522455	1106130	11322922
**C10**	379.235	11.95	0	0	2214029	416746.9	3751452	6845523	7817874	2751325	14185844	15109440	24095188	15482888	18703299	54420306	533318.8	10709629	15108338	19607852	18105805	13730474	558993.4	15650748
**C11**	516.129	12.68	89981502	0	0	0	0	0	0	0	0	0	0	0	0	0	0	0	0	0	0	0	0	0
**C12**	984.457	14.95	1.2E+08	0	0	0	0	0	0	0	0	0	0	0	0	0	0	0	0	0	0	0	0	0
**C13**	411.227	15	0	15579370	3180262	3404221	14897505	10231683	12205185	6985392	7187333	15386951	13774365	7567501	68708163	17332193	1533545	66877027	7393143	46499145	49921194	34238974	5058204	50821902
**C14**	449.259	15.67	0	65334579	796187.1	3441752	0	0	0	0	934760.1	1062303	2361872	1365957	2890385	7904423	0	0	243983.2	0	0	16783363	0	4611131
**C15**	468.325	15.81	94260032	0	0	0	0	0	0	0	0	0	0	0	0	0	0	0	0	0	0	0	0	0
**C16**	838.4	17.16	1.16E+08	0	0	0	0	0	0	0	0	0	0	0	0	0	0	0	0	0	0	0	0	0
**C17**	838.398	17.73	47624680	0	0	0	0	0	0	0	0	0	0	0	0	0	0	0	0	0	0	0	0	0
**C18**	260.18	18.2	0	72235170	10320454	35838284	15364805	16631743	14422911	11134194	11025568	15246951	11353482	10091405	31702543	11261621	35695357	39539922	3195353	10357391	14307817	49938195	44244871	48112428
**C19**	838.399	18.22	57928488	0	0	0	0	0	0	0	0	0	0	0	0	0	0	0	0	0	0	0	0	0
**C20**	470.342	18.43	2.68E+08	0	0	0	0	0	0	0	0	0	0	0	0	0	0	0	0	0	0	0	0	0
**C21**	646.369	18.47	1.34E+08	0	0	0	38243.29	52240.57	0	0	0	48994.17	260203.3	77755.21	0	65652.57	0	0	0	0	0	0	0	0
**C22**	260.178	19.05	0	1.02E+08	15137612	55001690	20822906	15717007	13227571	12653693	14373358	16268939	16233740	15471234	26328201	18965307	51886704	39709231	12465303	16899959	34905231	60506557	67365452	51681038
**C23**	176.087	19.96	0	2.81E+08	85839459	2.2E+08	1.34E+08	1.49E+08	1.3E+08	1.23E+08	1.23E+08	1.26E+08	1.08E+08	1.18E+08	1.46E+08	1.09E+08	2.28E+08	1.94E+08	95584041	1.21E+08	1.92E+08	2.34E+08	2.65E+08	2.38E+08
**C24**	276.172	19.96	0	2.2E+08	45245642	1.51E+08	81433713	92721896	74441321	70505888	69866751	71821468	58901393	67315356	94163740	61711907	1.68E+08	1.43E+08	53379064	72608931	1.38E+08	1.79E+08	1.97E+08	1.84E+08
**C25**	452.328	19.97	83316494	0	2352559	0	4931597	5492244	0	0	0	4537020	3560946	4034915	0	3530007	0	0	0	0	0	6183421	0	0
**C26**	470.345	19.99	45517922	4827411	1955435	3981447	3850787	4342637	3567821	3499088	3303540	3419148	2842735	3167998	3701740	2637861	4048041	4851261	2642784	3346549	5144274	4975251	4369761	5007308
**C27**	824.42	20.49	93452923	0	0	0	0	0	0	0	0	0	0	0	0	0	0	0	0	0	0	0	0	0
**C28**	549.349	20.79	0	1.3E+08	614021.9	12136235	0	0	0	0	539151.1	819193	2025280	898167.8	6125213	7807853	0	0	162284.8	159964.3	1209242	26145927	0	7972426
**C29**	283.231	21.03	0	91941681	4702317	6564121	0	1188607	698351.1	1012422	512647.9	1389321	1185460	778911.3	5589739	3925017	0	7945391	3725044	2323726	18304544	15480760	1627363	17596538
**C30**	375.249	22.37	62025568	36335498	57853358	69579472	38924114	39871126	37164242	45422106	36404202	50327990	36565531	36940521	60383243	34876998	2.7E+08	65174851	34276970	35101580	42351872	34392654	42405941	33631743
**C31**	824.419	22.65	43214814	0	0	0	0	0	0	0	0	0	0	0	0	0	0	0	0	0	0	0	0	0
**C32**	283.229	22.85	0	98092554	10759909	15928608	207575.9	1033473	769069.6	711656.8	1103186	2533353	2515834	2005261	6176516	8014798	0	12692411	3425646	2474686	36151913	57014881	3537766	38172518
**C33**	531.334	23.14	0	1.92E+08	34811657	25196002	982574.3	634886.8	1022424	291857.8	2633889	3173918	5729365	4756579	22302730	48709325	0	14335719	2197311	2331188	16337040	1.51E+08	0	70808488
**C34**	545.314	23.68	0	97337630	14377386	13357193	0	485191.8	785926.3	214110.4	1788033	2490823	3455840	2986248	8474297	19246688	0	10674362	1061387	1583100	15673893	73584042	639916.6	38985464
**C35**	531.34	24.02	0	1.12E+08	2928170	8449185	0	0	0	0	389225.8	522524.4	1170324	696708.8	3369712	7288457	0	572866.5	266841.3	220153.6	2189066	30163225	0	8171939
**C36**	324.138	24.75	1.26E+08	0	0	0	0	0	0	0	0	0	0	0	0	0	0	0	0	0	0	0	0	0

**C37**	304.202	25.65	0	1.29E+08	9279752	88201053	18332906	40254774	27335203	28507460	24855424	31290394	22772425	23363836	50919938	21588717	89853400	95777167	13594589	25927536	80523082	1.14E+08	1.43E+08	1.23E+08
**C38**	176.082	25.65	0	1.29E+08	10436114	96152985	20790297	45969675	31064174	32315177	28607357	34982339	26473840	26258778	54356217	23180024	88734254	95580471	14908783	27560057	81851725	1.12E+08	1.45E+08	1.24E+08
**C39**	296.235	25.89	4164794	0	4177520	4334570	6089933	6890995	7640776	8331233	8922407	8766936	9181360	9664153	8440032	9536648	73696389	8074979	10517920	9589997	7081599	5543144	5721551	5741112
**C40**	547.329	25.9	0	77508165	7760676	5352370	93260.76	275916.8	258328.3	130776.4	927135.1	1355793	1901618	1522803	6804617	9771553	479348.9	8160250	434478.8	839588.3	7065540	56872895	0	24387240
**C41**	605.407	26.57	0	68843352	1270817	8099797	434334.5	312782.2	240009.1	206402	248355.7	320880.9	348352.1	252631.7	3223727	0	0	921033.4	268808.2	406138.9	1857119	11480085	820534.1	4012002
**C42**	136.055	26.58	0	3.1E+08	1.89E+08	2.54E+08	1.4E+08	1.14E+08	1.03E+08	91695489	1.03E+08	1.24E+08	1.29E+08	1.06E+08	2.01E+08	1.51E+08	1.96E+08	2.55E+08	1.03E+08	1.33E+08	2.35E+08	2.76E+08	2.62E+08	3.03E+08
**C43**	392.2	27.62	76308092	0	0	0	0	0	0	0	0	0	0	0	0	0	0	0	0	0	0	0	0	0
**C44**	491.341	27.7	0	3.8E+08	54571458	1.89E+08	2386008	6885163	7258128	3821953	9204962	19460149	19772400	13308289	43397693	30895506	11750475	1.01E+08	7848093	7414018	1.17E+08	1.96E+08	1.07E+08	1.62E+08
**C45**	320.201	29.09	0	85700349	11421470	38380634	17413801	5633946	3941061	5390801	5360321	6478311	6145391	5971896	16591035	8404537	45246700	44712734	7377967	6292492	37894093	45848357	44838274	45561772
**C46**	547.33	29.49	0	91181561	7567954	5169735	98793.85	0	286176.8	172749.2	946202.6	1500424	2075421	1599745	7995980	10598608	0	10099648	465779.8	821950.3	7850185	61857669	0	26562580
**C47**	586.354	29.53	0	4819895	145512.3	9354199	143082.6	294845.6	140153.5	165553.9	105573.9	162866.4	167544	120555.1	1041753	198549.7	42967290	859898.4	120103.1	229570.5	973421.8	899562.6	9325579	1715835
**C48**	290.152	29.63	0	70784648	12206652	16951123	4759409	24814139	8899738	17770309	5769981	11177466	7159581	5980176	59767593	7046978	1.39E+08	1.02E+08	4414312	15450271	74070897	65610847	68282307	76220676
**C49**	118.078	29.97	0	0	0	0	0	0	0	0	0	0	0	0	2.08E+08	4016298	4021614	24725267	2406605	4179202	19330949	13163655	9801541	6982069
**C50**	565.415	30.06	0	0	1330085	0	0	0	0	0	12856967	28768142	27107340	25494190	0	5284885	0	0	2308432	0	11026057	1.91E+08	0	1.21E+08
**C51**	575.364	30.26	0	46954903	4609040	2547511	0	187610.2	225816.3	181540.8	465130.8	734163.7	915468.3	729152.1	3760961	4279708	1809057	6427744	355728.2	653777.4	3426961	30570336	329555.9	14037648
**C52**	519.38	30.71	0	56343884	6235864	35387885	0	647850.3	0	462125.7	866909.7	1987900	2108114	1246738	5077060	3399084	2812877	16726125	591403.1	615788.9	19930264	30270274	23919279	23421781
**C53**	332.235	30.84	286461.6	1.6E+08	13820531	1.2E+08	20248711	60288471	39706547	44771512	34736211	47489374	34400433	32614708	85636163	34261689	1.37E+08	1.37E+08	19025969	40330016	1.13E+08	1.43E+08	1.82E+08	1.54E+08
**C54**	176.082	30.84	0	1.19E+08	8217487	89373175	13139956	40425767	26016630	29455475	23058241	31152257	22259415	20941296	57008556	21151753	96874822	98698838	11719082	24993833	77052910	1.01E+08	1.36E+08	1.09E+08
**C55**	601.381	30.93	0	94396163	10712349	11887718	0	318255.6	344119.8	0	720442.2	1258060	1503358	1158421	7321376	10044528	1234541	12399695	302161.9	697127.4	9742348	58377116	680705.1	32195026
**C56**	470.34	31.19	57038738	0	0	0	0	0	0	0	0	0	0	0	0	0	0	0	0	0	0	0	0	0
**C57**	136.054	32	0	1.56E+08	72443076	1.11E+08	16957553	19614289	15184300	14690800	15623240	25320234	26190982	18071692	75813089	33729155	38474773	1.23E+08	13546078	24404557	84893783	1.13E+08	1.05E+08	1.48E+08
**C58**	419.355	33.27	635341	81916868	5112102	3096767	663118.4	1033222	663211	396789.3	289228	415186.7	929455	658828	6181878	2814711	1742483	14517398	2340399	1373519	31394461	25183800	2908143	26936370
**C59**	547.409	33.61	0	1.02E+08	11567083	59996489	0	965547.9	793159.9	648755.2	1180975	3008941	2999007	1704583	12106865	5405566	5213327	37218039	609399.4	972178.1	39492733	52666195	46985034	42702375
**C60**	575.365	34.35	0	59972159	4577292	3290425	0	173306.4	144968	130291.3	442489.7	766681	981871.9	767387.7	4541872	4466142	328973.4	7532096	146848.4	428170.2	3909397	37658547	650369.4	16675313
**C61**	603.395	34.86	0	1.2E+08	12975196	7566995	0	200400.6	190400.7	206449.3	870727.4	1625419	1783523	1186743	12567507	10537139	1227790	22431706	247819.4	887550	10536871	95026741	728248.9	44472572
**C62**	332.236	37.04	0	1.18E+08	51413630	86511593	7167184	11578996	8611091	9108562	8035690	15147869	14620901	10021184	61065796	21775721	38671051	1.03E+08	7300000	14969915	59760264	88400356	76943954	1.05E+08
**C63**	426.28	37.14	0	2.29E+08	70220321	1.55E+08	9733143	36031135	20828139	18738205	13781351	23528407	16812809	13654700	57963719	7484472	50470215	1.92E+08	12969412	25469508	2.08E+08	1.99E+08	2.33E+08	2.76E+08
**C64**	346.212	38.83	0	5664906	677667.4	979114.3	0	1010970	313244.7	1343977	319754.7	598421.5	357393.5	346680.9	6475988	321332.3	1.28E+08	15270552	254961.8	784030.6	3947278	4058390	7022360	4144530
**C65**	603.395	39.39	0	1.44E+08	17162884	8229190	0	230048.2	267207.2	194097.3	935221.9	1778517	2277468	1668276	19165093	12208708	1590870	33272245	287376.8	1044854	13469091	1.09E+08	739128.4	57647722
**C66**	346.217	39.47	0	27730595	8164005	2665012	547529.8	7549510	2435764	6664973	1496917	4007480	2088699	1656290	43759548	1994725	77897246	74304084	892826	5287299	28673908	30188055	36090817	42312120
**C67**	903.607	41.7	0	0	0	0	0	0	0	0	0	0	0	0	7940467	0	1.18E+08	6594862	0	0	0	5206966	1620243	3532045
**C68**	481.342	45.17	0	94375132	27221098	77092361	1714735	13205578	5393119	7165055	4401001	9890954	5429074	4497092	36315975	2984333	38255059	1.22E+08	2907021	8644337	93348709	84665351	1.14E+08	1.39E+08

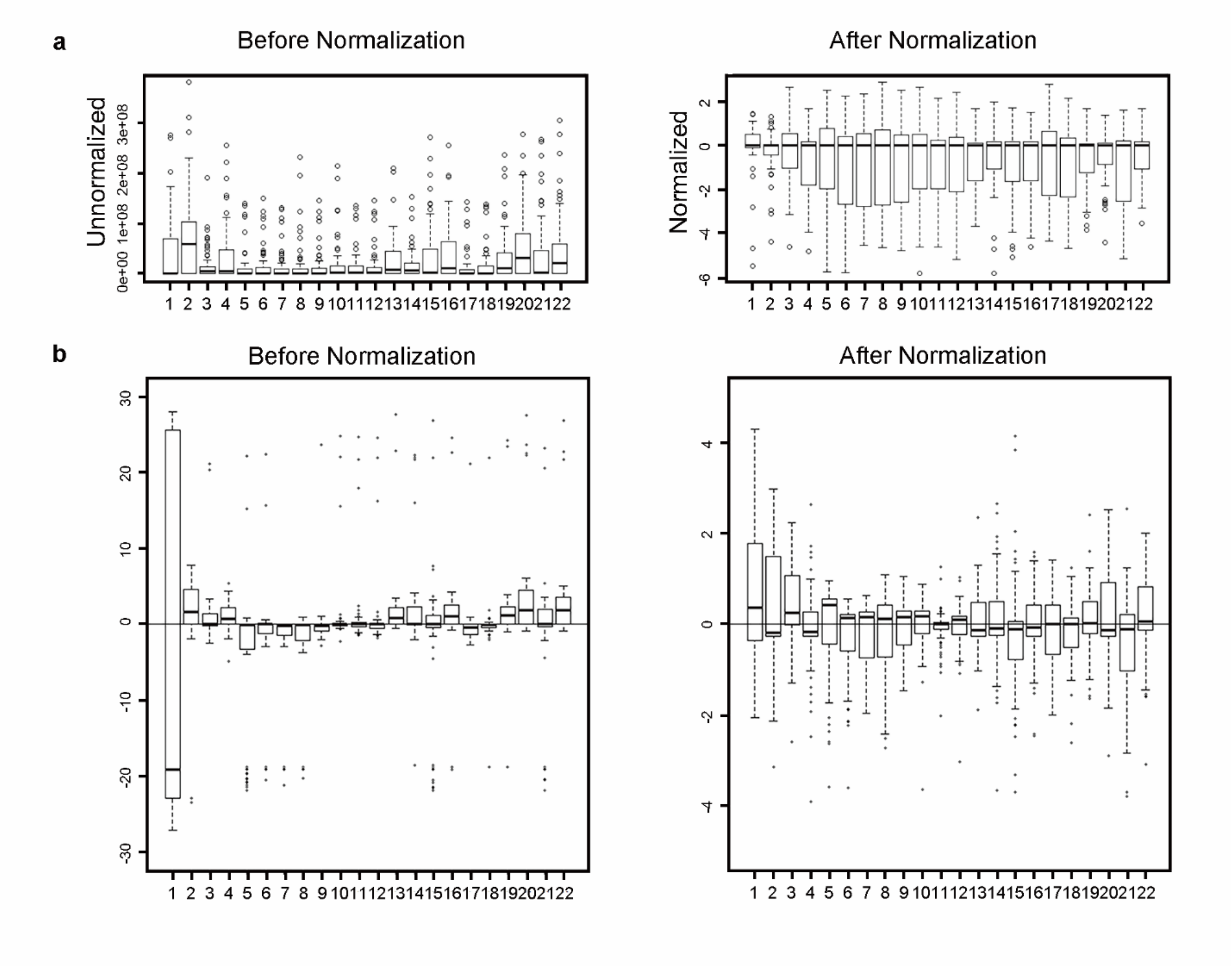

Supplementary Figure S3. Data distribution before and after normalization. **(a)** Data distribution of peak area of components in 22 ginger samples. **(b)** The relative log abundance of peak area in 22 ginger samples. Boxplots were created in RStudio.

Supplementary Table S3. The most effective components and their effects on the proliferation of TNBC cells.

	**Mass**	**RT**	**MDA-MB-231**	**HCC1806**	**MDA-MB-468**
**C42**	136.055	26.58	Increase IC_{50}		
**C38**	176.082	25.65		Decrease IC_{50}	
**C54**	176.082	30.84		Decrease IC_{50}	
**C22**	260.178	19.05	Decrease IC_{50}	Increase IC_{50}	Increase IC_{50}
**C18**	260.18	18.2	Decrease IC_{50}	Decrease IC_{50}	Increase IC_{50}
**C39**	296.235	25.89			Decrease IC_{50}
**C37**	304.202	25.65		Decrease IC_{50}	
**C45**	320.201	29.09		Increase IC_{50}	Increase IC_{50}
**C36**	324.138	24.75		Increase IC_{50}	Increase IC_{50}
**C53**	332.235	30.84		Decrease IC_{50}	
**C43**	392.2	27.62	Increase IC_{50}	Increase IC_{50}	
**C14**	449.259	15.67	Decrease IC_{50}		Increase IC_{50}
**C15**	468.325	15.81	Increase IC_{50}	Increase IC_{50}	
**C56**	470.34	31.19	Increase IC_{50}	Increase IC_{50}	
**C20**	470.342	18.43		Increase IC_{50}	Increase IC_{50}
**C26**	470.345	19.99			Increase IC_{50}
**C11**	516.129	12.68	Increase IC_{50}	Increase IC_{50}	
**C2**	543.213	7.32		Increase IC_{50}	Increase IC_{50}
**C8**	555.213	10.38		Increase IC_{50}	Increase IC_{50}
**C6**	562.17	10.14	Increase IC_{50}	Increase IC_{50}	
**C50**	565.415	30.06		Decrease IC_{50}	
**C1**	578.165	7.05	Increase IC_{50}	Increase IC_{50}	
**C3**	675.255	7.47		Increase IC_{50}	Increase IC_{50}
**C31**	824.419	22.65	Increase IC_{50}	Increase IC_{50}	
**C27**	824.42	20.49	Increase IC_{50}	Increase IC_{50}	
**C17**	838.398	17.73	Increase IC_{50}	Increase IC_{50}	
**C19**	838.399	18.22	Increase IC_{50}	Increase IC_{50}	
**C16**	838.4	17.16		Increase IC_{50}	Increase IC_{50}
**C12**	984.457	14.95		Increase IC_{50}	Increase IC_{50}

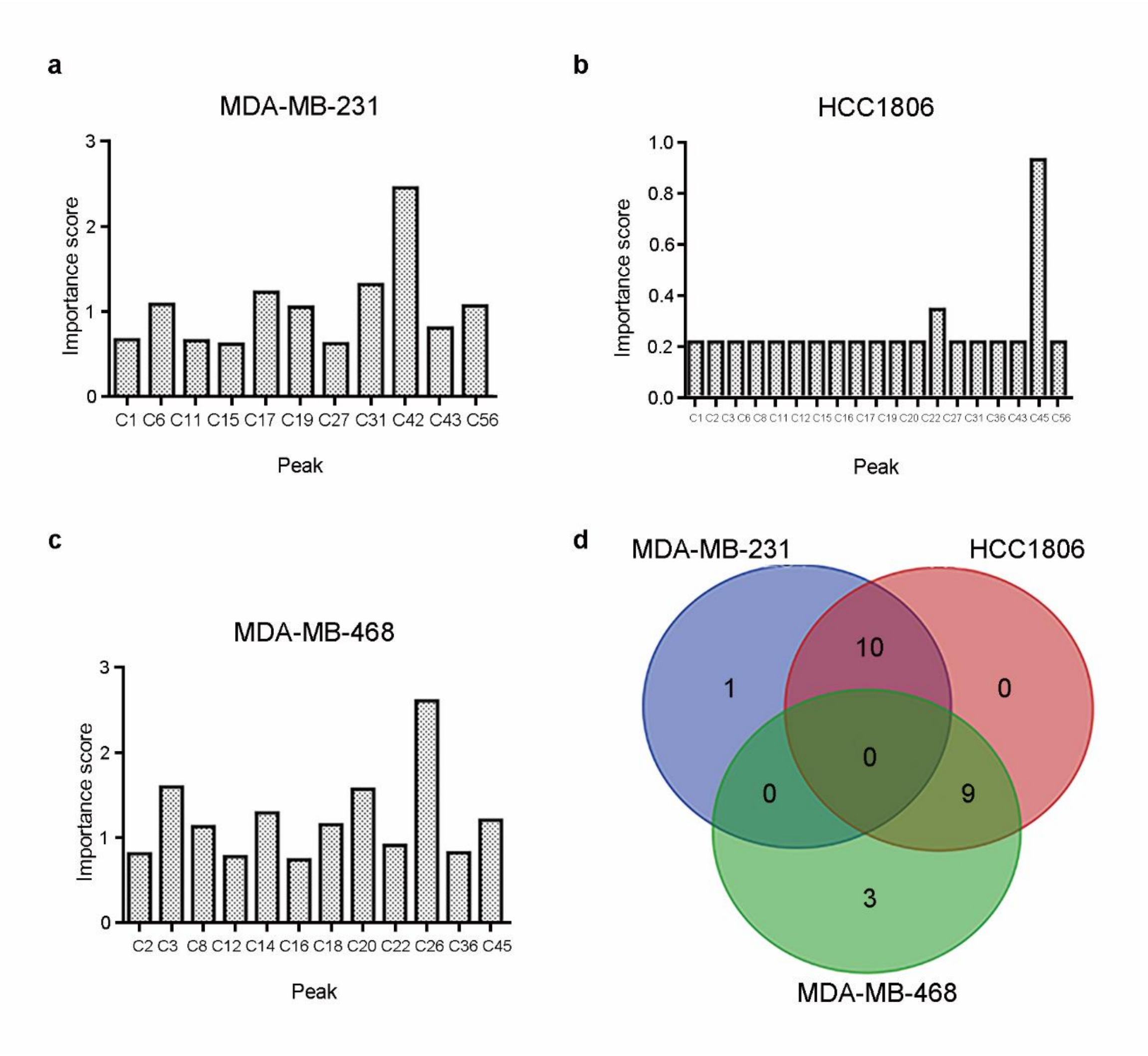

Supplementary Figure S4. The components which increased anti-proliferative effects of 22 ginger sample extracts. (**a-c**) The importance score of analytes found to be the most effective in increasing the IC_{50} values against MDA-MB-231 **(a)**, HCC1806 **(b)**, and MDA-MB-468 **(c)**, respectively. **(d)** The Venn diagram to show the components found in decreasing anti-proliferative effects of 3 TNBC cell lines.

Supplementary Table S4. The possible suggestions of compound identity for analytes of interest.

Peak No.	Mass	RT	Importance Score			Possible Compound	
			MDA-MB-231	HCC1806	MDA-MB-468	Possible Compound 1	Possible Compound 2
**C14**	449.259	15.67	-4.279		1.3	PE (phosphoethanolamine)	PC (phosphocholine)
**C18**	260.18	18.2	-0.912	-0.61	1.158	6-Paradol	
**C22**	260.178	19.05	-0.022	0.35	0.917	6-Paradol	
**C38**	176.082	25.65		-0.487		Ethyl cinnamate	Cinnamyl acetate
**C37**	304.202	25.65		-0.476		8-Shogaol	8-Gingerol
**C39**	296.235	25.89			-1.877	Pinolenic Acid	
**C50**	565.415	30.06		-1.507		PCs	
**C54**	176.082	30.84		-0.622		Cinnamyl acetate	Ethyl cinnamate
**C53**	332.235	30.84		-0.563		10-Gingerol	

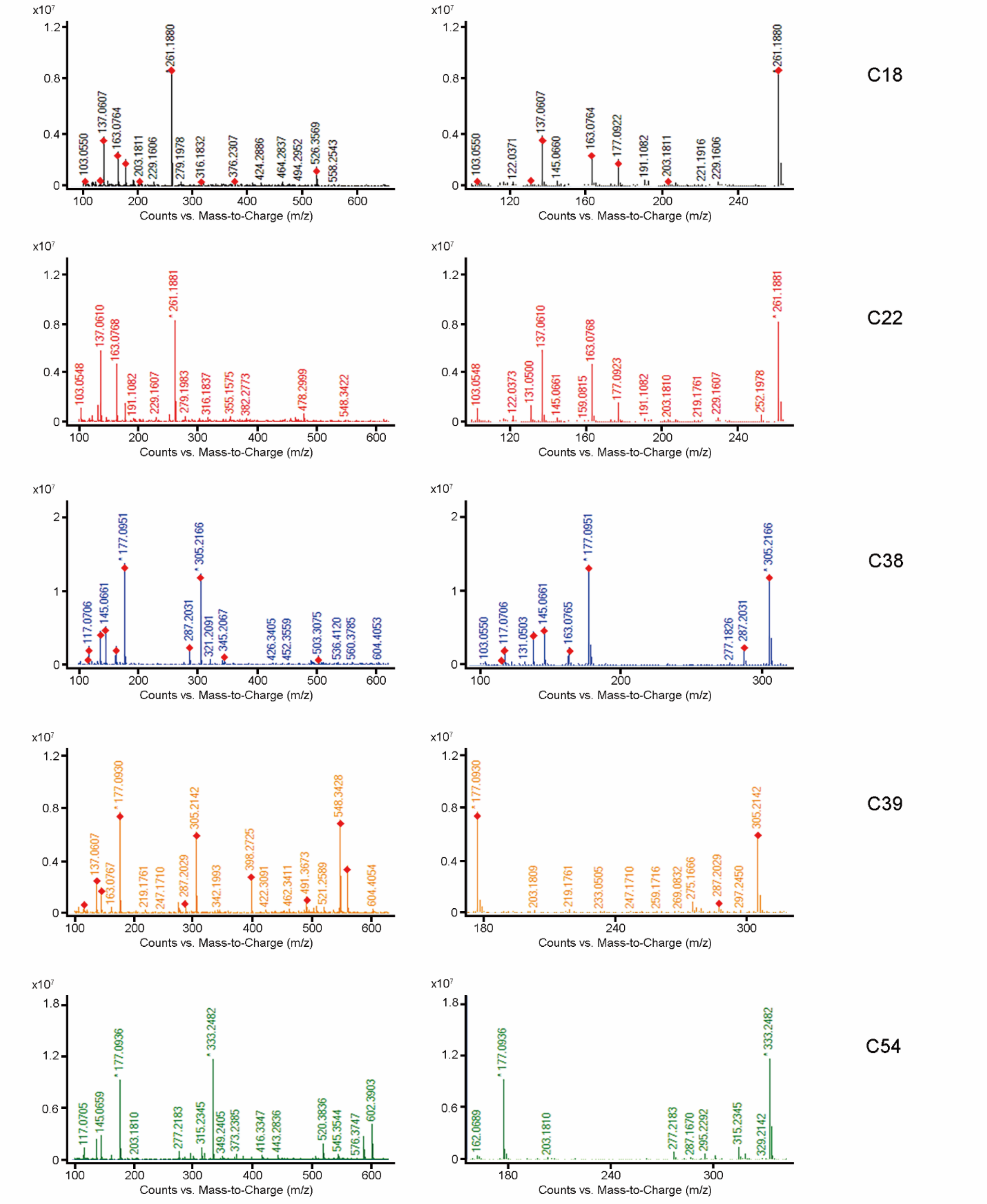

Supplementary Figure S5. The positive MS/MS spectrum information of components which increased anti-proliferative effects but cannot be confirmed by standards.

Supplementary Table S5. The absolute quantification for 8-gingerol and 10-gingerol in all 22 ginger samples.

Ginger sample	8-gingerol			10-gingerol		
	Peak area	UV abundance	Quantification (μg/mg)	Peak area	UV abundance	Quantification (μg/mg)
**1**	0	ND*	ND	286461.6	ND	ND
**2**	129000000	1989.82	0.79	160000000	3801.71	3.02
**3**	9279752	47.1	0.02	13820531	ND	ND
**4**	88201053	509.49	0.20	120000000	949.17	0.75
**5**	18332906	ND	ND	20248711	ND	ND
**6**	40254774	133.56	0.05	60288471	157.96	0.13
**7**	27335203	75.79	0.03	39706547	93.83	0.07
**8**	28507460	ND	ND	44771512	108.4	0.09
**9**	24855424	ND	ND	34736211	111.03	0.09
**10**	31290394	95.49	0.04	47489374	167.81	0.13
**11**	22772425	ND	ND	34400433	ND	ND
**12**	23363836	ND	ND	32614708	107.41	0.09
**13**	50919938	205.32	0.08	85636163	360.73	0.29
**14**	21588717	ND	ND	34261689	159.58	0.13
**15**	89853400	653.9	0.26	137000000	1216.87	0.97
**16**	95777167	503.83	0.20	137000000	1023.88	0.81
**17**	13594589	ND	ND	19025969	ND	ND
**18**	25927536	ND	ND	40330016	103.58	0.08
**19**	80523082	335.82	0.13	113000000	937.67	0.75
**20**	114000000	812.93	0.32	143000000	1543.47	1.23
**21**	143000000	1331.64	0.53	182000000	2198.7	1.75
**22**	123000000	910.62	0.36	154000000	1743.8	1.39

*ND: Not detected.
